# On the Impact of Digitalization and Artificial Intelligence on Employers' Flexibility Requirements in Occupations—Empirical Evidence for Germany

**DOI:** 10.3389/frai.2022.868789

**Published:** 2022-05-03

**Authors:** Anja Warning, Enzo Weber, Anouk Püffel

**Affiliations:** ^1^Department Forecasts and Macroeconomic Analyses, Institute for Employment Research (IAB), Nuremberg, Germany; ^2^Institute of Economics and Econometrics, Universität Regensburg, Regensburg, Germany; ^3^Universität Regensburg, Regensburg, Germany

**Keywords:** digitalization, artificial intelligence, flexible working conditions, occupations, employer survey

## Abstract

Artificial intelligence (AI) has a high application potential in many areas of the economy, and its use is expected to accelerate strongly in the coming years. This is linked with changes in working conditions that may be substantial and entail serious health risks for employees. With our paper we are the first to conduct an empirical analysis of employers' increasing flexibility requirements in the course of advancing digitalization, based on a representative business survey, the IAB Job Vacancy Survey. We combine establishment-level data from the survey and occupation-specific characteristics from other sources and apply non-linear random effects estimations. According to employers' assessments, office and secretarial occupations are undergoing the largest changes in terms of flexibility requirements, followed by other occupations that are highly relevant in the context of AI: occupations in company organization and strategy, vehicle/aerospace/shipbuilding technicians and occupations in insurance and financial services. The increasing requirements we observe most frequently are those concerning demands on employees' self-organization, although short-term working-time flexibility and workplace flexibility also play an important role. The estimation results show that the occupational characteristics, independently of the individual employer, play a major role for increasing flexibility requirements. For example, occupations with a larger share of routine cognitive activities (which in the literature are usually more closely associated with artificial intelligence than others) reveal a significantly higher probability of increasing flexibility demands, specifically with regard to the employees' self-organization. This supports the argument that AI changes above all work content and work processes. For the average age of the workforce and the unemployment rate in an occupation we find significantly negative effects. At the establishment level the share of female employees plays a significant negative role. Our findings provide clear indications for targeted action in labor market and education policy in order to minimize the risks and to strengthen the chances of an increasing application of AI technologies.

## Introduction

Increasing digitalization, including the development and use of artificial intelligence (AI), has substantially changed working conditions in establishments and administrations. This is one of the main results obtained in the empirical analyses conducted by Warning and Weber (2018) and Warning et al. (2020) on the basis of data from a representative German employer survey. The analyses show, among other things, that employers with digitalization activities—including the application of artificial intelligence—specify higher flexibility requirements with respect to place of work, working time, and self-organization for their newly hired employees significantly more frequently compared to employers without digitalization activities.

As far as we know, that study was one of the first to deal with changes in qualitative working conditions in the course of digitalization. To date, most analyses from labor market research focus on the quantitative effects, and the debate surrounding whether digitalization and its components creates or suppresses employment remains in the foreground (DeCanio, 2016; Arntz et al., 2017, 2020; Acemoglu and Restrepo, 2020a).

Yet, serious research from both labor and health economics and sociology point to the possible negative effects of precisely that type of qualitative changes reported by Warning and Weber (2018) and Warning et al. (2020). According to that research, changing requirements of employers with regard to working place, working time and work organization are not regarded as positive by all employees, and digitalization causes a significant proportion of individual psychological stress (Diebig et al., 2020; Hartwig et al., 2020). In Germany almost half of all employees (46%) associate digitalization with an increasing workload, while only 9% experience a reduction of their workload (Institut DGB Index Gute Arbeit, 2016).

Health insurance providers, in turn, report an increase in illnesses related to such increasing workloads, deadlines and time pressures, as well as changing working hours, and warn of the negative health effects of digitalization, see for Germany Marschall et al. (2017). The increase in stress-related illnesses is not only associated with lost hours of work and a strain on health and social security funds, employers must also expect significant reductions in the performance of those who continue to work despite illness (Diebig et al., 2020).

Sociological research intensively discusses the possible effects of increasing flexibility in working-time. It can entail considerable negative aspects for workers if they face the challenge of reconciling changing working times with other areas of their life, which is not always possible without conflict and is not always cost neutral (Allen et al., 2000; Ford et al., 2007; Dettmers et al., 2013; Brough et al., 2020). Of course, other individuals benefit from more time flexibility in their jobs in terms of work-life balance, particularly when increasing flexibility goes hand in hand with a high level of individual freedom, rather than increasing control over what employees do minute by minute.

Potential negative effects have been documented in a large number of studies and are likely to be relevant in most areas of digitalization. Not least due to the challenges in the wake of the COVID-19 pandemic, the dynamics of digitalization processes have accelerated enormously and AI is gaining importance in modern economies (Brynjolfsson et al., 2018; Al Momani et al., 2021; Amankwah-Amoah et al., 2021). As is discussed by Warning and Weber (2018), establishments and administrations first develop their internal and external digitalization technologies and networks, whereas artificial intelligence is integrated at a later date, so far in only a minority of establishments. However, its speed of dissemination is strongly increasing and a broader discussion of the effects on employees—besides the question of whether jobs are being created or destroyed—is needed to counteract at an early stage any negative developments that might burden not only individuals, but also businesses and society. In doing so, we consider it highly important to take account of the specificities of occupations, since, as has already been discussed in the literature, the applications of AI may differ considerably between occupations and fields of activity (see section Available Research on AI and the Labor Market), which in turn may have an impact on the respective working conditions.

With our analyses we make a substantive empirical contribution to the discussion surrounding qualitative changes in working conditions in the course of digitalization and the use of AI, with a special focus on the role of occupation-specific characteristics. On the basis of data from a large, representative German employer survey we shed light on employers' changing flexibility demands regarding their employees' place of work, short-term changes in their working time and requirements regarding their self-organization. As far as we know, there is no other representative study available in this context, based on concrete assessments by a large number of employers in all industries and establishment sizes. Germany is a country with a strong digital development and high investments in the development and application of AI (OECD, 2020). Therefore, the results presented here are also highly relevant for other advanced economies and contribute to discussions at the European level dealing with changing working conditions.

Our article is structured as follows: Section Available Research on AI and the Labor Market provides an overview of the research conducted to date on labor market changes related to artificial intelligence, which so far mainly comprises research on potential quantitative effects. Section Method presents the data that we use for our study, explains the transformation of the data into a panel data set and justifies the selection of a non-linear random effects estimator. This part is followed by a description of some of the digital developments in Germany and of the occupations that are relevant in the context of AI applications in section Some Descriptive Results. Section Estimation Results discusses the results of the random effects estimations and the factors that emerge as relevant for employers' increasing requirements regarding their employees' flexibility in terms of their place of work, their working time and their self-organization. We summarize our results in section Discussion and Outlook and provide an outlook for future empirical research on the qualitative labor market effects of AI.

## Available Research On AI and The Labor Market

As is the case for digitalization in general, there is no unique definition of AI that expresses the diversity and breadth of both the technology and its potential applications, although we do not yet know all of the potential AI applications. Therefore, labor market researchers currently address above all the possible labor market effects of AI, while the actual labor market effects remain largely unknown, with little empirical work conducted on the topic so far.

Current research deals partly with conceptual boundaries and the ways that AI can be operationalized for empirical research (Ernst et al., 2019; Acemoglu and Restrepo, 2020b; Tolan et al., 2021). Building on or parallel to this, empirical work has also been conducted on the quantitative effects of AI on employment, wages, hires, and fluctuation (Felten et al., 2019; Webb, 2020; Georgieff and Hyee, 2021; Fossen and Sorgner, 2022). These quantitative studies have to make assumptions about how certain capabilities and tasks are changed by the application of AI technologies, which have to be defined initially, for example on the basis of interviews with experts from the AI field. The aim is to assess how the characteristics of occupations change with regard to the tasks to be performed and the skills required and to estimate the quantitative effects resulting from these changes. Research on changing tasks and the shifting importance of specific task types (types of manual and cognitive tasks) is usually a crucial element of these approaches.

For instance, in German labor market research, occupations are distinguished according to five task types (Spitz-Oener, 2006), see Table 1 for a description and examples. Using this concept Genz et al. (2021) discuss the idea of different stages of digital development that include AI in the youngest stage. They find that establishments that are active in this youngest stage (“4.0 adopters”) have a comparatively larger share of employees performing routine cognitive tasks in their job activities (36%), followed by non-routine analytical tasks and non-routine manual tasks. The degree of complexity involved in the job increases with ongoing digitalization, as does demand for IT staff (AI specialists, IT security consultants, cloud engineers) and staff in business services.

**Table 1:** Task types of occupations and examples.

**Task-type**	**Description**	**Occupations with highest shares in the task-type**
Non-routine analytical activities	Doing research, analyze, evaluate, plan, construct, design, develop rules/regulations, apply and interpret rules	Members of Parliament, Ministers, Architects, Civil Engineers, Veterinarians, Publicists
Interactive non-routine activities	Negotiate, represent interests, coordinate, organize, teach or train, sell, buy, advertise, entertain, present, employ or manage clients	Interpreters, translators, sales representatives, employment and professional advisers, consumer advisers
Cognitive routine activities	Calculate, make bookkeeping, correct text/data, measure length/height/temperature	Chemical laboratory technician, radio operator, data typist, telecommunication assembler
Non-routine manual activities	Repair or renovation of houses/flats/machinery/vehicles, restoration of art/monuments, service or accommodation of guests	Paving, earthmoving machine drivers, machine cleaners, railway drivers
Routine manual activities	Operating or controlling machines, equipping machines	Rubber converters, metal pullers, leather manufacturers, sheet metal presses

*Sources: Spitz-Oener (2006) p. 243, Dengler et al. (2014) p. 38*.

From the available studies, it can be deduced that AI is mainly used in occupational fields involving a high proportion of cognitive and analytical tasks. In these fields, based on a large amount of data, AI can strengthen the basis for decision-making by making it possible to systematically monitor and evaluate processes, thereby supporting people in their decision-making. In some areas AI can also take over the control of processes entirely. On the other hand, AI is used less in areas in which people interact strongly, as not all elements of human behavior can be replaced by technological systems.

The OECD recently published an article reviewing what is known about the labor market effects of AI, showing the potential of AI on the one hand and our very limited knowledge about the real labor market effects on the other hand (Lane and Saint-Martin, 2021). This applies in particular to knowledge about changing working conditions and employers' changing demands regarding flexibility, what might be even more important than in previous stages of digitalization. The authors provide an example of this for the case of AI-supported robots: Such robots might take over activities that are dangerous or physically very strenuous for humans, which has clear positive effects on the tasks to be performed, as they become less dangerous and less strenuous. However, if the humans have to adapt their work intensity and rhythm to the robot in a close human-machine-interaction, the work pressure might simultaneously increase and the freedom of action may decrease, leading to increasing stress and growing dissatisfaction, in turn causing (new) psychological stress for the employee. Another open issue in the context of AI is the availability of big data, which enables employers to closely monitor employees' activities and to steer these activities automatically in the short term. This not only raises questions concerning data protection and personal rights, but in practice pressurizes employees to respond at short notice to adaptations intended by the AI system and to avoid any mistakes and misconduct while carrying out work.

## Method

### Establishment Data From the IAB Job Vacancy Survey

In the study presented here, we examined the role of occupation- and establishment-specific characteristics for increasing flexibility requirements expressed by employers.

We took up some of the findings obtained by Warning and Weber (2018) on significant changes in working conditions and again use the IAB Job Vacancy Survey (JVS) for our new approach. The JVS is a representative employer survey conducted at regular intervals among employers in Germany. Its overall aim is to determine the current demand for labor and to observe staff-search and hiring processes in detail (Davis et al., 2014; Bossler et al., 2020). Every year some 12,000 establishments and administrations of all sizes and from all sectors of the economy complete the written questionnaire in the fourth quarter of the year. (According to the sampling method, the term “establishment” always refers to establishments and public administrations with at least one employee covered by social security contributions.) The information they report on vacancies, employment, and the development of search and hiring processes are extrapolated to all establishments and all new hires in Germany, thereby providing a unique, representative picture of the current labor market development in Germany (on the extrapolation, see Brenzel et al., 2016). The JVS is quality assured in accordance with the regulations laid down by the European Commission concerning the collection, measurement and calculation of job vacancy and employment data that are gathered in this survey and are officially published by Eurostat in the context of labor demand data for the European countries (Eurostat, n. d).

**Figure 1:**
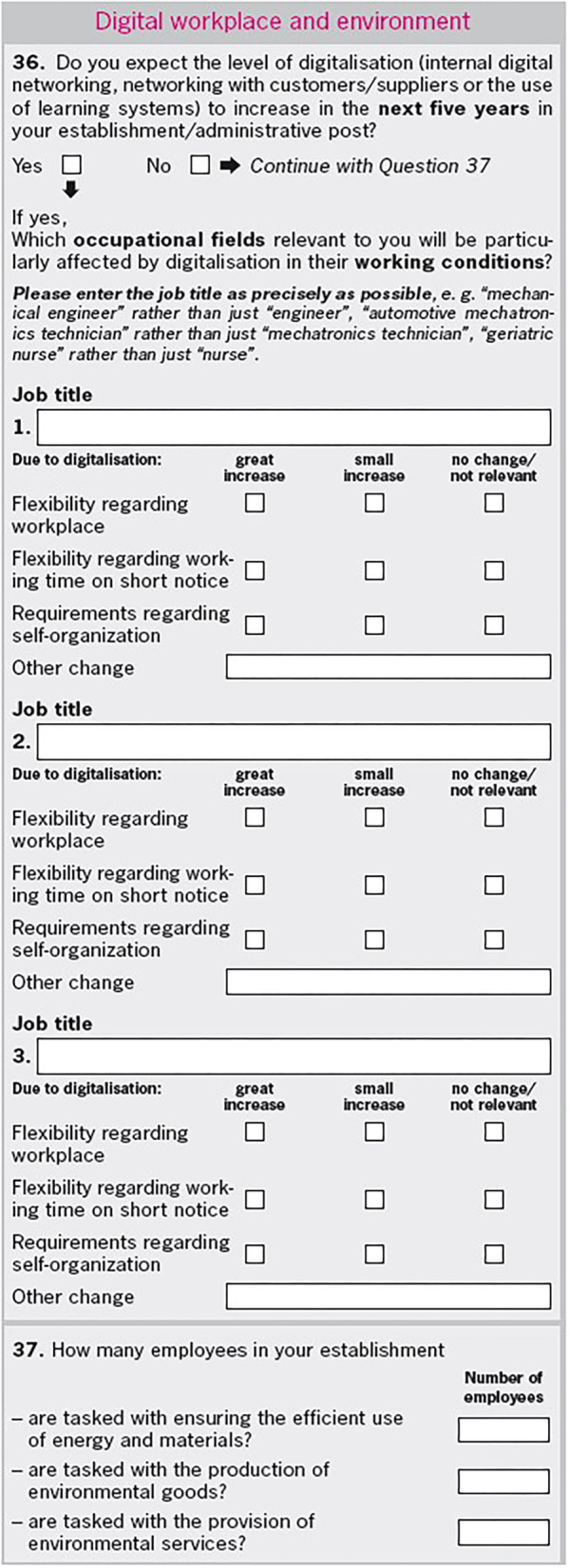
IAB Job Vacancy Survey 2016, written questionnaire, p. 5.

In 2016 we integrated new detailed questions into a special questionnaire section of the JVS. It focused on changing flexibility requirements in occupations by those employers who expect increasing digitalization in the subsequent 5 years, see Figure 1. In the first question (question 36 in the JVS) the participating establishments, or their managers or personnel managers, are asked whether their particular establishment is expecting an increase in digital development over the following 5 years. As in the previous analysis of Warning and Weber, digital development is defined as internal digital networking, networking with customers/suppliers and the use of learning systems. Learning systems as part of artificial intelligence systems are thus included in our study.

All establishments that answer the first question with YES (a total of 4,262 establishments) are then asked to report the occupations for which they expect particularly strong changes in employees' qualitative working conditions as a result of increasing digitalization. The questionnaire gives the possibility to state a maximum of three occupations. The changes in working conditions refer to flexibility in terms of workplace, flexibility regarding working time on short notice and demands regarding employees' self-organization. The wording in the special questionnaire section deliberately refers only to (great and small) increasing or unchanging flexibility requirements, because our research focuses only on increases, not decreases.

Restricting the number of occupations that establishments could mention here to a maximum of three was a compromise: On the one hand, we wanted to investigate positive changes in flexibility requirements by individual occupations. On the other hand, an already extensive written survey like the JSV cannot be extended by too many additional questions, as this may lead to a drop in establishments' willingness to participate, thereby endangering the success of the entire survey. However, the restriction to three occupations proved in retrospect to be very meaningful and does not lead to a distortion of the results: The vast majority of those establishments expecting an increase in digitalization provided detailed information on flexibility requirements for one or two occupations. Only rarely did an establishment report three occupations in the questionnaire. Therefore the answers reflect employers' assessments of the occupations that they consider to be most strongly affected, this has to be taken into account when interpreting the survey results.

For the subsequent estimations we calculated three new binary variables from the JVS data. They are independent of each other and are the dependent variables in our models:

1) increasing requirements regarding flexibility in terms of place of work,
2) increasing requirements regarding short-term flexibility in working time and
3) increasing demands regarding self-organization.

Each binary variable took the value 1 if the establishment reported a small or large increase in the flexibility required in the specific occupation. It took the value 0 if the establishment indicated no change or no relevance of this requirement.

In addition to the data on changing requirements by occupation we utilized standard establishment-specific structural data from the JVS. They describe the establishment's individual employment and labor demand situation that might affect the employer's individual decisions regarding the flexibility required of their employees. Specifically, we used information on region and workforce size, the share of academics, the share of employees with vocational qualifications and the share of women. We included data on the establishment's overall labor demand, such as the expected employment development, the number of new hires, job vacancies as a proportion of employment and the fluctuation in the particular economic sector. We also included data on the existence of a works council and collective agreements, as this might hinder or delay the implementation of new technologies and the associated changes in working conditions (Warning and Weber, 2018). Table 2 provides a descriptive overview of all establishment-specific variables used in our models.

**Table 2:** Descriptives of the variables used in the estimation models.

**Variables**	**Mean**	**Std. dev**.	**Min**	**Max**
**Dependent variables**				
Work place flexibility	0.0428	0.2023	0	1
Short-term flexibility in working time	0.0632	0.2433	0	1
Demands on self-organization	0.0775	0.2674	0	1
**Independent variables**				
**Occupation-specific:**				
Share of interactive non-routine activities	10.8945	12.0753	0.214	39.199
Share of non-routine analytical activities	19.6029	12.2761	4.098	51.101
Share of non-routine manual activities	24.6106	20.4172	0.619	57.080
Share of routine cognitive activities	28.7483	16.5478	8.978	59.502
Average age of employees	41.1220	1.6945	38.613	45.523
Employment growth rate 2013–2016	8.2091	3.8719	1.642	15.325
Fluctuation rate in 2016	2.0818	1.1458	0.403	3.927
Unemployment rate in 2016	7.2371	3.8274	2.447	15.985
**Establishment-specific:**				
Region	0.5622	0.4961	0	1
Size class	1.9315	0.5377	1	3
Labor-turnover rate by sector	65.3976	39.1471	27.3	152.1
Expected employment trend	1.7320	0.6170	1	3
New employees hired in the previous year	0.7842	0.4114	0	1
Vacancies as a proportion of total employment	5.2215	14.1550	0	200
Collective agreement in place	0.4940	0.5000	0	1
Existence of works council	0.3071	0.4613	0	1
Share of skilled workers	65.0450	30.0866	0	100
Share of academics	17.9145	24.8894	0	100
Share of women	41.7667	27.3834	0	100

*Source: IAB Job Vacancy Survey 2016, Data Warehouse of the Federal Employment Agency 2019, own calculations*.

### Data on Occupation-Specific Characteristics

In order to be able to depict occupation-specific characteristics in the best possible way, we added various occupation-specific variables that are independent of the individual establishments. First, we used information on the shares of five task types in each occupational group (Spitz-Oener, 2006). Data for the year 2016 come from IAB task research, providing the shares of non-routine analytical, non-routine interactive, routine cognitive, non-routine manual, and routine manual activities in each occupation (Dengler et al., 2014). Table 1 provides a description of these types, as well as examples of occupations that have a relatively large share of the respective task type.

Second, we used structural information from the Federal Employment Agency related to the occupational group: the average age of the workforce, the employment growth rate between 2013 and 2016, the labor turnover rate in 2016 and the unemployment rate in 2016. These data allow us to describe general differences between the occupational groups as precisely as possible, thereby minimizing the risk of omitted variables in our estimation models. Table 2 contains a descriptive overview of the occupation-specific variables.

### Creation of a Panel Dataset for Random Effects Estimations

The reported occupations were originally coded according to the German Classification of Occupations 2010 at the 4-digit level (Statistical Offices of the Federation and the Länder, n. d). To ensure that the number of cases per occupational unit was sufficiently high for the analyses, we aggregated the original data at the level of 14 occupational groups and finally obtained a dataset containing information on changing requirements in 14 occupational groups from about 4,200 establishments.

In order to take into account heterogeneity effects and to analyze increasing flexibility requirements in the context of occupations, we transformed this original cross-sectional dataset into a panel data structure. This allows the use of a panel data model, we specifically chose the non-linear random effects model (Cameron and Trivedi, 2010; Wooldridge, 2010). A fixed effects model would not yield estimates for the occupation-specific variables which are the focus of our interest (see next paragraph on these variables). Besides that argument, fixed effects models do not function in the specific case of our data structure. This is characterized by the peculiarity that the three binary dependent variables have a relatively high number of zeros and a relatively low number of ones, meaning that there is relatively little variation in the dependent variables by 14 occupational groups and about 4,200 establishments. As a result, the estimation coefficients (see section Estimation Results) are small, but as is shown with the parameter rho in the estimations in Tables 5–7, a standard pooled estimation would lead to inconsistent parameter estimates and a panel data estimation is the preferred approach here.

## Some Descriptive Results

### Digital Development in German Establishments

The following results are weighted with the standard weighting factors calculated for the data of the IAB Job Vacancy Survey. The figures in Tables 3, 4 thus represent the total numbers of the respective establishments in the economy.

**Table 3:** Sectors of the economy with the respective shares of companies that expect increasing digitalization over the next 5 years.

Financial services, Insurance	63%
Liberal professions, scientific and technical services	50%
Machines, electronics, vehicles	41%
Information and communication	41%
Public administration	39%
Health and social services	36%
Education, child care	34%
Trade, retail, repairs	33%
Other services	31%
Chemistry, plastics, glass, construction materials	31%
Energy utilities	30%
Transport, warehouses	30%
Metals, metal production	29%
Nutrition, textiles, clothing, furniture, etc.	27%
Water, waste management	26%
Real estate	26%
Other commercial services	25%
Agriculture, forestry	24%
Wood, paper, printing	24%
Construction	18%
Hospitality	18%
Art, entertainment, recreation	15%
Mining, ores and earths	13%

*Source: IAB Job Vacancy Survey 2016, own calculations, weighted results*.

**Table 4:** Number of establishments with positive expectations of increasing flexibility requirements in the respective occupation.

**Occupational field**	**Number of establishments expecting a change in working conditions in the occupational field**	**Number of establishments in which changing working conditions are accompanied by increasing demands of the following types:**		
		**Increasing workplace flexibility**	**Increasing temporal flexibility**	**Increasing self-organization**
Office and secretarial	57,738	19,401	41,721	53,081
Company organization and strategy	34,467	21,207	27,654	32,328
Vehicle manufacture, aerospace, shipbuilding technicians	32,220	14,534	15,420	22,641
Insurance and financial services	31,589	19,419	24,773	28,445
Tax consultancy	27,475	15,101	15,791	24,794
Purchasing and sales	27,232	19,839	23,203	24,051
Construction planning and supervision, architecture	23,965	10,245	15,676	19,753
Accounting, controlling and auditing	18,629	11,289	14,720	16,843
Public administration	17,499	8,745	11,276	15,247
Mechanical engineering and operating technology	14,734	8,167	12,662	13,129

*Source: IAB Job Vacancy Survey 2016, own calculations, weighted results*.

A total of 4,262 establishments in the survey expected increasing digitalization in the following 5 years. Altogether, they represent 700,000 establishments in the German economy, which is equivalent to a share of about 32%. The highest shares by economic sector are found in financial and 256 insurance services, at 63%, followed by liberal professions, scientific and technical services at 257 50%, see Table 3. The sectors with the lowest shares of establishments expecting an increase in digitalization include for instance art, entertainment and recreation, and hospitality.

Establishments with more than 250 employees are more likely to expect increasing digitalization than medium-sized and small ones. On the whole our results are similar to those obtained in other studies on the spread of digitalization in Germany (Reimann et al., 2020).

### Occupations and Increasing Flexibility Requirements

Table 4 shows a list of the most frequently mentioned occupations and the number of establishments with positive digitalization expectations and positive expectations regarding increasing flexibility requirements in these occupations. Office and secretarial occupations were mentioned most frequently, by about 58,000 establishments and administrations, followed by three occupations that are highly relevant in the context of artificial intelligence: occupations in company organization and strategy (34,000), vehicle/aerospace/shipbuilding technicians (32,000) and occupations in insurance and financial services (32,000).

The table reveals the high relevance of changes in employees' self-organization during the course of digitalization: In all the occupations listed there, this kind of flexibility requirement was mentioned most often by the employers, followed by increasing temporal flexibility and increasing workplace flexibility. As we know, digitalization and in specific the introduction of artificial intelligence systems are closely linked to changes in working structures (Quelle). Our results on the special relevance of increasing demands regarding self-organization underline this statement.

## Estimation Results

### Occupational Characteristics

Tables 5–10 show the coefficients and marginal effects calculated from our three random effects estimations. In the following we use the marginal effects as the basis for the discussion of our findings, see Table 11 for a comparison between the models. The effects are small in quantitative terms, which is due to the characteristics of the data structure (see section Method). Nevertheless, the effects are highly meaningful, as is confirmed by both the error probabilities and the quality criteria of our estimations.

**Table 5:** Estimation results: increasing requirements regarding workplace flexibility.

	**Coefficient**		**Std. err**.	**95% Confidence** **interval**	
**Occupation-specific:**					
Share of interactive non-routine activities	0.01017		0.00856	−0.00662	0.02696
Share of non-routine analytical activities	−0.01822		0.01046	−0.03871	0.00227
Share of non-routine manual activities	0.00886		0.00779	−0.02413	0.00641
Share of routine cognitive activities	0.03691	^***^	0.00687	0.02344	0.05037
Average age of employees	0.11902	^**^	0.05593	−0.22864	−0.00940
Employment growth rate 2013–2016	0.16675	^***^	0.04629	−0.25747	−0.07603
Fluctuation rate in 2016	0.49464	^***^	0.16873	0.16394	0.82533
Unemployment rate in 2016	0.07555	^**^	0.03171	−0.13771	−0.01340
**Establishment-specific:**					
Region (east)	0.08300		0.05018	−0.01536	0.18136
Establishment size class (<10)					
10–249	0.14026		0.07369	−0.28469	0.00418
>250	0.01945		0.11307	−0.20217	0.24106
Labor-turnover rate by sector	0.00014		0.00066	−0.00115	0.00143
Expected employment trend (constant)					
Increasing	0.08414		0.05562	−0.02488	0.19315
Decreasing	0.25326	^***^	0.08202	0.09251	0.41402
New employees hired in the previous year	0.10874		0.06711	−0.02280	0.24028
Vacancies as a proportion of total employment	0.00281		0.00164	−0.00041	0.00603
Collective agreement in place	0.06956		0.05580	−0.17893	0.03980
Existence of works council	0.00414		0.06666	−0.12652	0.13480
Share of skilled workers	0.00181		0.00112	−0.00038	0.00401
Share of academics	0.00372	^***^	0.00129	0.00119	0.00625
Share of women	0.00416	^***^	0.00093	−0.00598	−0.00234
Constant	1.48933		2.22000	−2.86178	5.84044
Rho	0.01507	^***^	0.00862	0.00488	0.04558

*Source: IAB Job Vacancy Survey 2016, own calculations with a random effects estimation*,

*p < 0.1;

** *p < 0.05*;

*** *p < 0.01*.

**Table 6:** Estimation results: increasing requirements regarding short-term flexibility in working time.

	**Coefficient**		**Std. err**.	**95% Confidence** **interval**	
**Occupation-specific:**					
Share of interactive non-routine activities	0.00593		0.00794	−0.00963	0.02149
Share of non-routine analytical activities	0.02591	^***^	0.00937	−0.04428	−0.00755
Share of non-routine manual activities	0.01332		0.00716	−0.02735	0.00071
Share of routine cognitive activities	0.03651	^***^	0.00628	0.02421	0.04881
Average age of employees	0.15857	^***^	0.05051	−0.25757	−0.05956
Employment growth rate 2013–2016	0.15568	^***^	0.04296	−0.23988	−0.07149
Fluctuation rate in 2016	0.47546	^***^	0.15443	0.17278	0.77814
Unemployment rate in 2016	0.07443	^***^	0.02871	−0.13070	−0.01817
**Establishment-specific:**					
Region (east)	0.04496		0.04179	−0.03696	0.12688
Establishment size class (<10)					
10–249	0.04996		0.06386	−0.07521	0.17512
>250	0.17310		0.09624	−0.01553	0.36173
Labor-turnover rate by sector	0.00060		0.00056	−0.00170	0.00049
Expected employment trend (constant)					
Increasing	0.11208	^**^	0.04614	0.02164	0.20251
Decreasing	0.14334	^*^	0.07034	0.00547	0.28120
New employees hired in the previous year	0.06931		0.05608	−0.04060	0.17923
Vacancies as a proportion of total employment	−0.00101		0.00163	−0.00420	0.00217
Collective agreement in place	−0.07340		0.04648	−0.16450	0.01769
Existence of works council	0.03594		0.05527	−0.14427	0.07240
Share of skilled workers	0.00070		0.00091	−0.00107	0.00248
Share of academics	0.00077		0.00108	−0.00134	0.00289
Share of women	0.00304	^***^	0.00077	−0.00455	−0.00153
Constant	3.86104		2.01311	−0.08459	7.80667
Rho	0.01333	^***^	0.00686	0.00483	0.03619

*Source: IAB Job Vacancy Survey 2016, own calculations with a random effects estimation*,

* *p < 0.1*;

** *p < 0.05*;

*** *p < 0.01*.

**Table 7:** Estimation results: increasing requirements regarding self-organization.

	**Coefficient**		**Std. err**.	**95% Confidence** **interval**	
**Occupation-specific:**					
Share of interactive non-routine activities	0.01087		0.00968	−0.00811	0.02985
Share of non-routine analytical activities	0.02112		0.01106	−0.04279	0.00055
Share of non-routine manual activities	−0.00865		0.00861	−0.02553	0.00823
Share of routine cognitive activities	0.03422	^***^	0.00757	0.01938	0.04906
Average age of employees	0.14493	^**^	0.05673	−0.25613	−0.03374
Employment growth rate 2013–2016	0.15120	^***^	0.05115	−0.25145	−0.05096
Fluctuation rate in 2016	0.41074	^**^	0.18199	0.05405	0.76742
Unemployment rate in 2016	0.08282	^**^	0.03416	−0.14978	−0.01587
**Establishment-specific:**					
Region (east)	0.03579		0.03818	−0.03905	0.11062
Establishment size class (<10)					
10–249	0.09868		0.05886	−0.01668	0.21405
>250	0.16452		0.08867	−0.00927	0.33832
Labor-turnover rate by sector	0.00145	^**^	0.00052	−0.00247	−0.00044
Expected employment trend (constant)					
Increasing	0.05175		0.04221	−0.03097	0.13447
Decreasing	0.02201		0.06596	−0.10728	0.15129
New employees hired in the previous year	0.09557		0.05141	−0.00520	0.19634
Vacancies as a proportion of total employment	0.00064		0.00149	−0.00356	0.00228
Collective agreement in place	0.04365		0.04249	−0.12693	0.03964
Existence of works council	0.01638		0.05011	−0.11460	0.08183
Share of skilled workers	0.00089		0.00083	−0.00074	0.00252
Share of academics	0.00062		0.00099	−0.00132	0.00256
Share of women	0.00155	^**^	0.00070	−0.00292	−0.00019
Constant	3.44720		2.25166	−0.96598	7.86038
Rho	0.02101	^***^	0.00923	0.00883	0.04918

*Source: IAB Job Vacancy Survey 2016, own calculations with a random effects estimation*,

* p < 0.1;

** *p < 0.05*;

*** *p < 0.01*.

**Table 8:** Marginal effects: increasing requirements regarding workplace flexibility.

	**Marginal effect**		**Std. err**.	**95% Confidence** **interval**	
**Occupation-specific:**					
Share of interactive non-routine activities	0.00026		0.00022	−0.00017	0.00069
Share of non-routine analytical activities	−0.00047		0.00027	−0.00099	0.00006
Share of non-routine manual activities	−0.00023		0.00020	−0.00062	0.00016
Share of routine cognitive activities	0.00095	^***^	0.00018	0.00059	0.00130
Average age of employees	−0.00305	^**^	0.00142	−0.00583	−0.00027
Employment growth rate 2013–2016	−0.00427	^***^	0.00118	−0.00659	−0.00195
Fluctuation rate in 2016	0.01267	^***^	0.00430	0.00424	0.02109
Unemployment rate in 2016	−0.00193	^**^	0.00082	−0.00354	−0.00033
**Establishment-specific:**					
Region (east)	0.00213		0.00129	−0.00041	0.00466
Establishment size class (<10)					
10–249	−0.00370		0.00205	−0.00771	0.00031
>250	0.00055		0.00322	−0.00575	0.00686
Labor-turnover rate by sector	0.00000		0.00002	−0.00003	0.00004
Expected employment trend (constant)					
Increasing	0.00213		0.00143	−0.00067	0.00492
Decreasing	0.00696	^***^	0.00249	0.00208	0.01183
New employees hired in the previous year	0.00278		0.00173	−0.00060	0.00617
Vacancies as a proportion of total employment	0.00007		0.00004	−0.00001	0.00015
Collective agreement in place	−0.00178		0.00143	−0.00459	0.00103
Existence of works council	0.00011		0.00171	−0.00324	0.00345
Share of skilled workers	0.00005		0.00003	−0.00001	0.00010
Share of academics	0.00010	^***^	0.00003	0.00003	0.00016
Share of women	−0.00011	^***^	0.00002	−0.00015	−0.00006

*Source: IAB Job Vacancy Survey 2016, own calculations with a random effects estimation, margins at means*,

* p < 0.1;

** *p < 0.05*;

*** *p < 0.01*.

**Table 9:** Marginal effects: increasing requirements regarding short term flexibility in working time.

	**Marginal effect**		**Std. err**.	**95% Confidence** **interval**	
**Occupation-specific:**					
Share of interactive non-routine activities	0.00023		0.00030	−0.00037	0.00082
Share of non-routine analytical activities	−0.00099	^***^	0.00036	−0.00169	−0.00029
Share of non-routine manual activities	−0.00051		0.00027	−0.00105	0.00003
Share of routine cognitive activities	0.00140	^***^	0.00025	0.00091	0.00189
Average age of employees	−0.00607	^***^	0.00191	−0.00981	−0.00232
Employment growth rate 2013–2016	−0.00596	^***^	0.00164	−0.00917	−0.00274
Fluctuation rate in 2016	0.01819	^***^	0.00588	0.00666	0.02971
Unemployment rate in 2016	−0.00285	^***^	0.00110	−0.00501	−0.00068
**Establishment-specific:**					
Region (east)	0.00172		0.00160	−0.00142	0.00486
Establishment size class (<10)					
10–249	0.00186		0.00234	−0.00273	0.00645
>250	0.00682		0.00386	−0.00075	0.01439
Labor-turnover rate by sector	−0.00002		0.00002	−0.00007	0.00002
Expected employment trend (constant)					
Increasing	0.00430	^**^	0.00180	0.00076	0.00783
Decreasing	0.00558		0.00288	−0.00007	0.01122
New employees hired in the previous year	0.00265		0.00215	−0.00156	0.00687
Vacancies as a proportion of total employment	−0.00004		0.00006	−0.00016	0.00008
Collective agreement in place	−0.00281		0.00178	−0.00631	0.00069
Existence of works council	−0.00137		0.00212	−0.00552	0.00277
Share of skilled workers	0.00003		0.00003	−0.00004	0.00009
Share of academics	0.00003		0.00004	−0.00005	0.00011
Share of women	−0.00012	^***^	0.00003	−0.00018	−0.00006

*Source: IAB Job Vacancy Survey 2016, own calculations with a random effects estimation, margins at means*,

^*^ p < 0.1;

** *p < 0.05*;

*** *p < 0.01*.

**Table 10:** Marginal effects: increasing requirements regarding self-organization.

	**Marginal effect**		**Std. err**.	**95% Confidence** **interval**	
**Occupation-specific:**					
Share of interactive non-routine activities	0.00052		0.00046	−0.00039	0.00143
Share of non-routine analytical activities	−0.00101		0.00053	−0.00204	0.00003
Share of non-routine manual activities	−0.00041		0.00041	−0.00122	0.00039
Share of routine cognitive activities	0.00163	^***^	0.00038	0.00090	0.00237
Average age of employees	−0.00691	^**^	0.00271	−0.01222	−0.00161
Employment growth rate 2013–2016	−0.00721	^***^	0.00246	−0.01204	−0.00239
Fluctuation rate in 2016	0.01960	^**^	0.00870	0.00254	0.03665
Unemployment rate in 2016	−0.00395	^**^	0.00165	−0.00718	−0.00073
**Establishment-specific:**					
Region (east)	0.00171		0.00182	−0.00187	0.00528
Establishment size class (<10)					
10–249	0.00454		0.00264	−0.00064	0.00972
>250	0.00780		0.00429	−0.00061	0.01621
Labor-turnover rate by sector	−0.00007	^**^	0.00003	−0.00012	−0.00002
Expected employment trend (constant)					
Increasing	0.00248		0.00204	−0.00152	0.00648
Decreasing	0.00104		0.00314	−0.00512	0.00720
New employees hired in the previous year	0.00456		0.00247	−0.00028	0.00940
Vacancies as a proportion of total employment	−0.00003		0.00007	−0.00017	0.00011
Collective agreement in place	−0.00208		0.00203	−0.00606	0.00190
Existence of works council	−0.00078		0.00239	−0.00547	0.00391
Share of skilled workers	0.00004		0.00004	−0.00004	0.00012
Share of academics	0.00003		0.00005	−0.00006	0.00012
Share of women	−0.00007	^**^	0.00003	−0.00014	−0.00001

*Source: IAB Job Vacancy Survey 2016, own calculations with a random effects estimation, margins at means*,

* p < 0.1;

** *p < 0.05*;

*** *p < 0.01*.

**Table 11:** Comparison of the marginal effects of the three estimations.

	**Work place flexibility**		**Short term flexibility in working time**		**Demands on self-organization**	
**Occupation-specific:**						
Share of interactive non-routine activities	0.00026		0.00023		0.00052	
Share of non-routine analytical activities	−0.00047		−0.00099	^***^	−0.00101	
Share of non-routine manual activities	−0.00023		−0.00051		−0.00041	
Share of routine cognitive activities	0.00095	^***^	0.00140	^***^	0.00163	^***^
Average age of employees	−0.00305	^**^	−0.00607	^***^	−0.00691	^**^
Employment growth rate 2013–2016	−0.00427	^***^	−0.00596	^***^	−0.00721	^***^
Fluctuation rate in 2016	0.01267	^***^	0.01819	^***^	0.01960	^**^
Unemployment rate in 2016	−0.00193	^**^	−0.00285	^***^	−0.00395	^**^
**Establishment-specific:**						
Region (east)	0.00213		0.00172		0.00171	
Establishment size class (<10)						
10–249	−0.00370		0.00186		0.00454	
>250	0.00055		0.00682		0.00780	
Labor-turnover rate by sector	0.00000		−0.00002		−0.00007	^**^
Expected employment trend (constant)						
Increasing	0.00213		0.00430	^**^	0.00248	
Decreasing	0.00696	^***^	0.00558		0.00104	
New employees hired in the previous year	0.00278		0.00265		0.00456	
Vacancies as a proportion of total employment	0.00007		−0.00004		−0.00003	
Collective agreement in place	−0.00178		−0.00281		−0.00208	
Existence of works council	0.00011		−0.00137		−0.00078	
Share of skilled workers	0.00005		0.00003		0.00004	
Share of academics	0.00010	^*^ ^***^	0.00003		0.00003	
Share of women	−0.00011	^***^	−0.00012	^***^	−0.00007	^**^

*Source: IAB Job Vacancy Survey 2016, own calculations with a random effects estimation, margins at means*,

* *p < 0.1*;

** *p < 0.05*;

*** *p < 0.01*.

For all three kinds of flexibility requirements the share of routine cognitive activities is highly significant, with the highest value for increasing demands regarding self-organization. A one-percent increase in the share of routine cognitive activities raises the probability of increasing demands on self-organization by 0.16% points, the probability of increasing short-term working-time flexibility by 0.14% points and of increasing workplace flexibility by about 0.09% points. According to the literature occupations affected strongly by AI applications are often defined by relatively high shares of routine cognitive tasks or non-routine analytical tasks (Genz et al., 2021; Lane and Saint-Martin, 2021). Looking at the shares of routine cognitive activities in the occupational groups in Table 12, our estimates suggest this discussion with regard to occupations with a high share of routine cognitive activities: For instance, in business services and in business management and organization more than half of all tasks are routine cognitive tasks (59 and 56%, respectively). Here increasing digitalization, including the increasing use of AI, is more likely to be associated with employers demanding more flexibility, in particular with regard to self-organization and short-term flexibility in working time.

**Table 12:** Shares of task by occupational group 2016, as percentages.

	**Occupations in agriculture, forestry and horticulture**	**Manufacturing occupations**	**Occupations in manufacturing technology**	**Occupations in construction and building completion**	**Occupations in food and hospitality sector**	**Medical and non-medical health occupations**	**Social and cultural service occupations**	**Commercial occupations**	**Occupations in business management and organization**	**Occupations in business services**	**Occupations in IT and scientific services**	**Security occupations**	**Occupations in transport and logistics**	**Cleaning occupations**
Non-routine analytical tasks	15.4	7.4	18.1	14.6	8.7	19.5	36.2	12.6	31.4	22.2	51.1	22.6	10.4	4.1
Non-routine interactive tasks	2.6	0.7	1.0	0.4	12.3	17.1	39.2	34.3	10.8	17.5	8.8	5.8	1.8	0.2
Routine cognitive tasks	10.0	18.1	47.6	23.2	22.7	17.2	11.4	46.3	56.1	59.5	34.2	19.3	27.9	9.0
Routine manual tasks	34.3	64.7	22.0	13.2	17.2	4.1	2.0	3.2	1.1	0.0	5.2	0.9	28.4	29.6
Non-routine manual tasks	37.7	9.1	11.3	48.6	39.1	42.0	11.1	3.6	0.6	0.8	0.7	51.4	31.4	57.1

*Source: Calculation of the shares for the year 2016 by Dengler, K., Matthes, B. and Paulus, W. on basis of Dengler et al. (2014)*.

As the marginal effects show, the share of non-routine analytical tasks is negatively significant regarding increasing short-term flexibility in working time, it is not relevant regarding the other two types of flexibility. Looking at the examples of occupations with large shares of such non-routine analytical tasks in Table 12, this result is not surprising in the AI context. If AI is usable at all, it is used more as a supplementary technology. Human beings still have to make decisions and need to understand the AI technology and its applications. Specifically, the work involved in developing and implementing new AI technologies in the establishments may initially be very time-consuming and require a lot of attention from the people involved. It is necessary to understand in detail the interplay between technologies and humans, for which increasing requirements on short-term flexibility in working time, which workers often associate with increasing time pressure, is not a good basis.

Non-routine manual activities show no significant effects on the probability of increasing flexibility requirements. In the context of AI, as a special form of digital development, this result substantiates the discussions about the potential relevance of AI for certain occupations, but not for others.

In all three models, the average age of the employees in the occupational group is negatively and highly significantly related to increasing requirements, with the highest value regarding the demands for self-organization. This result is expectable and reflects the relatively high level of regulation of the German labor market, which protects older employees in many ways. The question also arises of whether older employees who are unwilling or unable to adapt to their employers' changing flexibility requirements are more likely to take up occupations with a lower (or slower) level of digital development or whether they are more frequently forced by their employers to change to other occupational fields or even to change the employer.

The occupation-related employment growth rate between 2013 and 2016, the period directly before the field period of the survey, shows a negative and highly significant value in all three models. An increase in the employment growth rate by 1% reduces the probability of increasing demands on self-organization by 0.7% points. Negative effects are also estimated for the unemployment rate. The fields of the labor market in which digital developments are particularly dynamic and where working conditions may change as a result are more likely to be those in which employers complain of worker and skills shortages. The unemployment rate is correspondingly low and workers' demands for a good work-life balance are likely to be correspondingly high. This is likely to limit employers' possibilities to further increase their flexibility requirements and may even force them to reduce their demands.

The fluctuation rate, i.e., the dynamics of entry and exit from employment in the respective occupational group, exhibits a significant positive effect in all models. High fluctuation means that a relatively large proportion of new employees are recruited relative to the existing workforce. Whereas in the case of the existing workforce, employers are dependent on employees' willingness to change and are not always able to implement changes with the scope and speed desired, in the case of new hires the employers can formulate the precise requirements and conditions that they consider to be in line with the new challenges and opportunities of digitalization. Effects on working conditions and flexibility requirements will therefore be more visible in the more dynamic occupational fields.

### Establishment Characteristics

In contrast to the occupational effects, the characteristics of the individual establishments play a minor role in explaining increasing flexibility requirements. The size of an establishment and the region in which it is located are not explanatory. Those operating in an industry with a high labor-turnover rate, and thus having to recruit and train new staff more often, are more likely to define increasing demands on employees' self-organization. This is not the case for the other two types of flexibility.

Positive employment expectations increase the probability of increasing demands for short-term flexible working hours. This is not true of the number of new hires in the previous year or of current vacancies as a proportion of the total workforce, (It should be taken into account that all the establishments in our estimates assume increasing digitalization over the next 5 years, see section Establishment Data From the IAB Job Vacancy Survey of this article).

The skill structure in the establishment shows no significance, except for the proportion of academics in model 1. Differences in skill levels are at least partly captured by the differences in the occupations. In our analyses differences at the occupational level are more relevant than differences at the establishment level.

The proportion of women in the workforce exhibits a significant negative marginal effect in all models. For instance, a one-percent increase in the share of female employees reduces the probability of increasing demands on short term flexibility in working time by 0.012% points. The possibilities for negotiation with female employees regarding increased workplace and short-term working-time flexibility are likely to be fewer than is the case with male employees, at least as far as employees with children or other caring responsibilities are concerned. In many families it is still the mothers who perform the majority of the care work and who have to reconcile this with their employment in terms of space and time. This means that they are tied to existing and stable agreements with their employers to a greater extent, which tends to oppose greater flexibility. The existence of a works council or collective agreements shows no effects in the three estimations.

## Discussion and Outlook

Our analyses contribute to the largely unexplored area of research on the qualitative effects of digitalization and the use of AI on working conditions, especially with regard to the demand for increasing flexibility in work assignments. We pay particular attention to the role played by differences between occupations, because, as is discussed in the literature, AI is affecting different occupational fields in different ways. To our knowledge, our study is the first one to present estimation results based on data from a large representative employer survey.

First of all, our study confirms some findings from previous literature on digitalization and AI: occupations for which employers expect the most substantial changes in working conditions as a result of digitalization include office and secretarial occupations as well as occupations in business organization. Occupations in vehicle, aerospace, space, and shipping technology and occupations in tax consulting are also frequently mentioned by the employers in the survey. According to the descriptive results, increasing requirements regarding workplace flexibility play a less significant role than short-term working-time flexibility and specifically the demands on employees' self-organization. These findings indirectly support the discussion surrounding the potential labor market effects of AI, according to which AI primarily changes work content and work processes, which is directly related to aspects of employees' self-organization. According to our results, the flexibility requirements are changing especially in those occupational fields that are undergoing particular strong changes in the context of AI, as discussed for instance by Lane and Saint-Martin (2021).

Using random effects estimations and including numerous establishment- and occupation-specific control variables, we show that it is above all the occupational and less the establishment-specific characteristics that determine the probability of employers demanding increasing flexibility. Increasing demands in terms of flexibility are particularly prevalent in occupational groups that involve a large proportion of routine cognitive activities. These are the fields that are likely to change more strongly with increasing use of AI.

The largest effect of the share of routine cognitive activities in quantitative terms is measured for the probability of increasing demands for employees' self-organization, again supporting arguments, that AI mainly changes work content and work processes. This is particularly important for public employment services: people seeking jobs in occupations with a large proportion of routine cognitive activities can be supported in a targeted manner with regard to their individual abilities and opportunities for a more flexible work engagement than they might be familiar with from previous jobs. This may concern skills in self-organization at work or advice about the advantages and disadvantages of more flexible working time. In fact, policy can focus on very specific areas of the labor market, because possible risks do not affect all occupational fields in which AI is used or might be relevant in future. In our estimations the proportion of manual tasks does not show any significant effect on the flexibility requirements. And occupations involving a large amount of interaction between employees are also less at risk of negative effects. Here, AI is likely to be used somewhat less, since interactions between people are more difficult to replace by machines.

Besides labor market policy also education policy plays a crucial role for the question of whether AI mainly has a negative impact on working conditions or not. Decisive possibilities for policy action are, for instance, the strategic development of the education and vocational training systems and the provision of a child care infrastructure that supports the reconciliation of a more flexible working and private life. For female employees in particular, the increasing use of AI and the associated demand for greater working-time flexibility is likely to be a major challenge and might even become an employment risk if adequate and flexible childcare facilities are not available.

Apart from the share of women, the establishment-specific characteristics play a subordinate role compared to the occupational characteristics. Employers see the challenge of compensating for additional individual burdens on employees in order to maintain the employees' productivity and job satisfaction, especially if the employers are to be increasingly threatened by labor shortages.

Future empirical research on the qualitative labor market effects of digitalization and AI should deal in depth with the role of certain occupations, which requires a larger number of cases in survey-based studies. How does AI change productivity on the one hand and individual stress on the other hand for different employee groups (female/male, young/old, employees with families/without families, etc.) in different occupational fields? Here the gender-related effects should be paid special attention in order to be able to counteract possible replacement effects at an early stage. What options exist for employers to compensate their employees for additional burdens, for example attractive holiday arrangements, further training opportunities, setting up long-term working time accounts with attractive conditions for the employee, through to financial compensation for increasing flexibility in work assignments? What are sustainable good and healthy working conditions that keep the workforce productive and satisfied in times of accelerating digitalization? The employer's perspective is important here for negotiating joint solutions, which makes a combination of both employer surveys and employee surveys highly attractive in this research field. Finally, international comparative analyses could take into account the specifics of different national labor market policies in the context of ongoing digitalization, which in general has been further accelerated by the current COVID-19 pandemic.

## Data Availability Statement

Publicly available datasets were analyzed in this study. This data can be found here: The Research Data Centre (FDZ) of the Federal Employment Agency at the Institute for Employment Research. https://fdz.iab.de/en.aspx.

## Author Contributions

All authors listed have made a substantial, direct, and intellectual contribution to the work and approved it for publication.

## Conflict of Interest

The authors declare that the research was conducted in the absence of any commercial or financial relationships that could be construed as a potential conflict of interest.

## Publisher's Note

All claims expressed in this article are solely those of the authors and do not necessarily represent those of their affiliated organizations, or those of the publisher, the editors and the reviewers. Any product that may be evaluated in this article, or claim that may be made by its manufacturer, is not guaranteed or endorsed by the publisher.
